# Supplementary figures and images for: Known allosteric proteins have central roles in genetic disease
Source: PLoS Comput Biol. 2022 Feb 9;18(2):e1009806. doi: 10.1371/journal.pcbi.1009806 (PMC10138267; doi:10.1371/journal.pcbi.1009806)

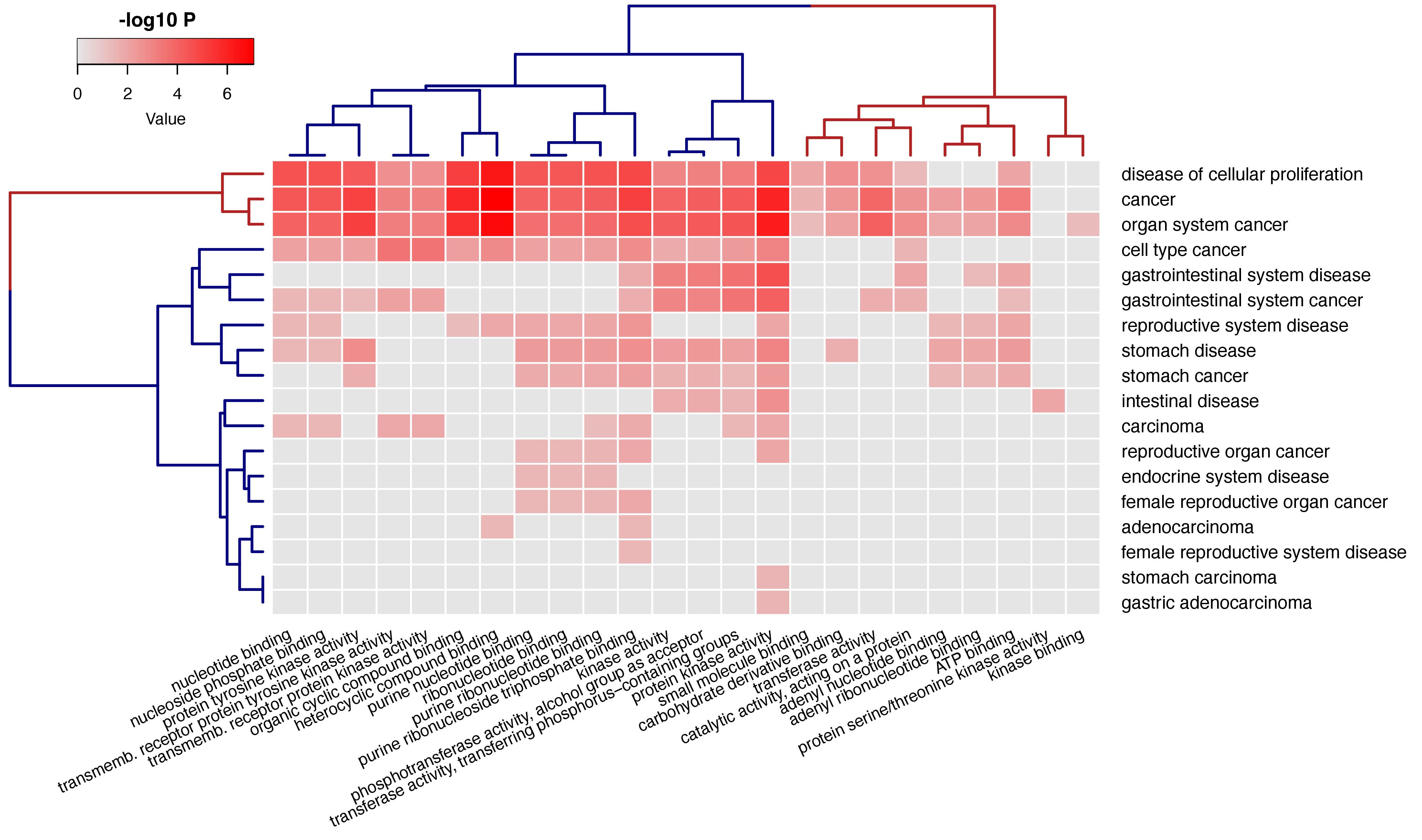

Supplement: S1 Fig — Most terms with significant overlaps are the result of associations between cancers and protein kinases. (TIF) [file pcbi.1009806.s001.tif]

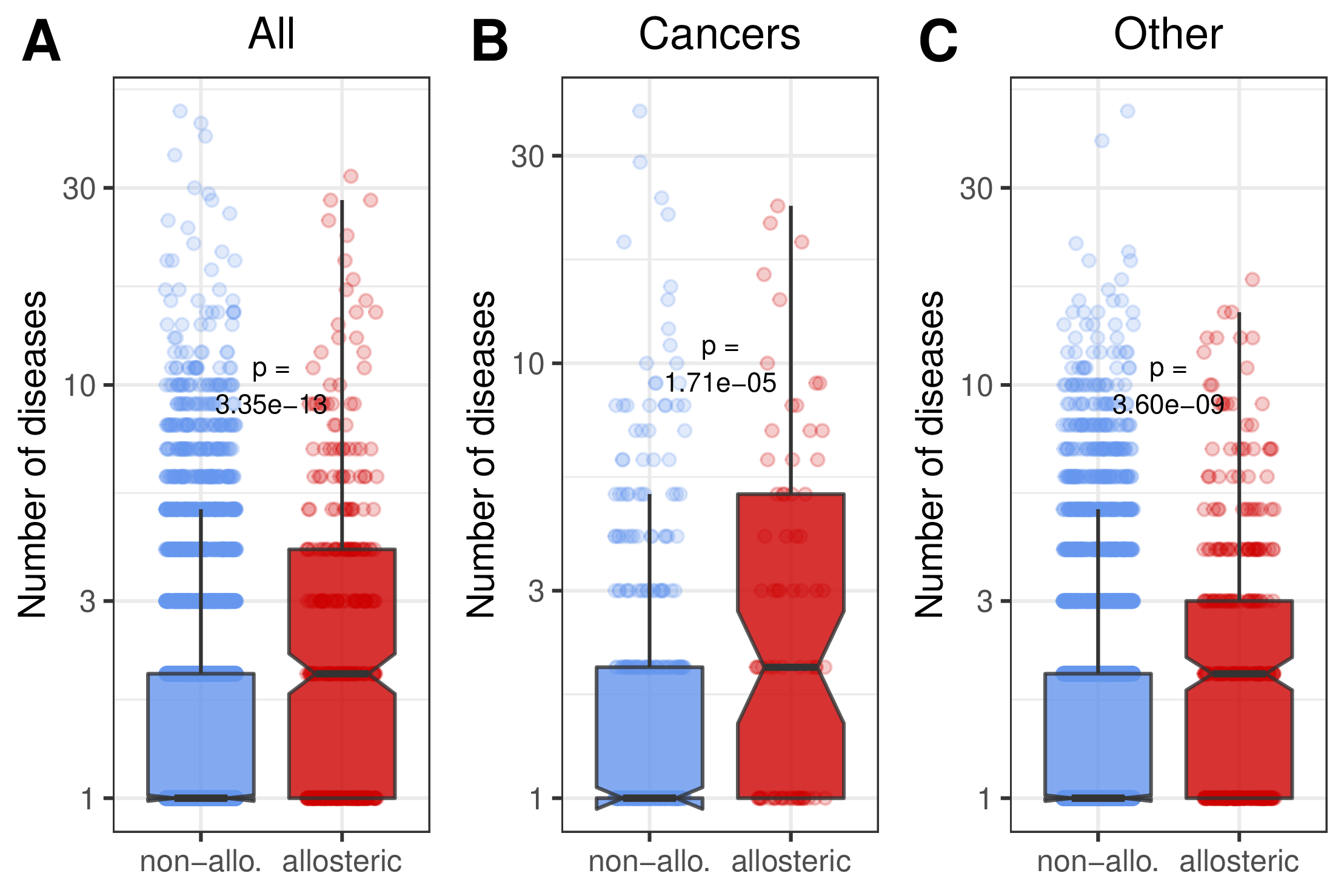

Supplement: S2 Fig — Allosteric proteins are associated with more diseases than non-allosteric ones (A), also when cancers (B), or non-cancerous diseases (C) are analysed separately. All statistical comparisons were made with Wilcoxon tests. (TIF) [file pcbi.1009806.s002.tif]

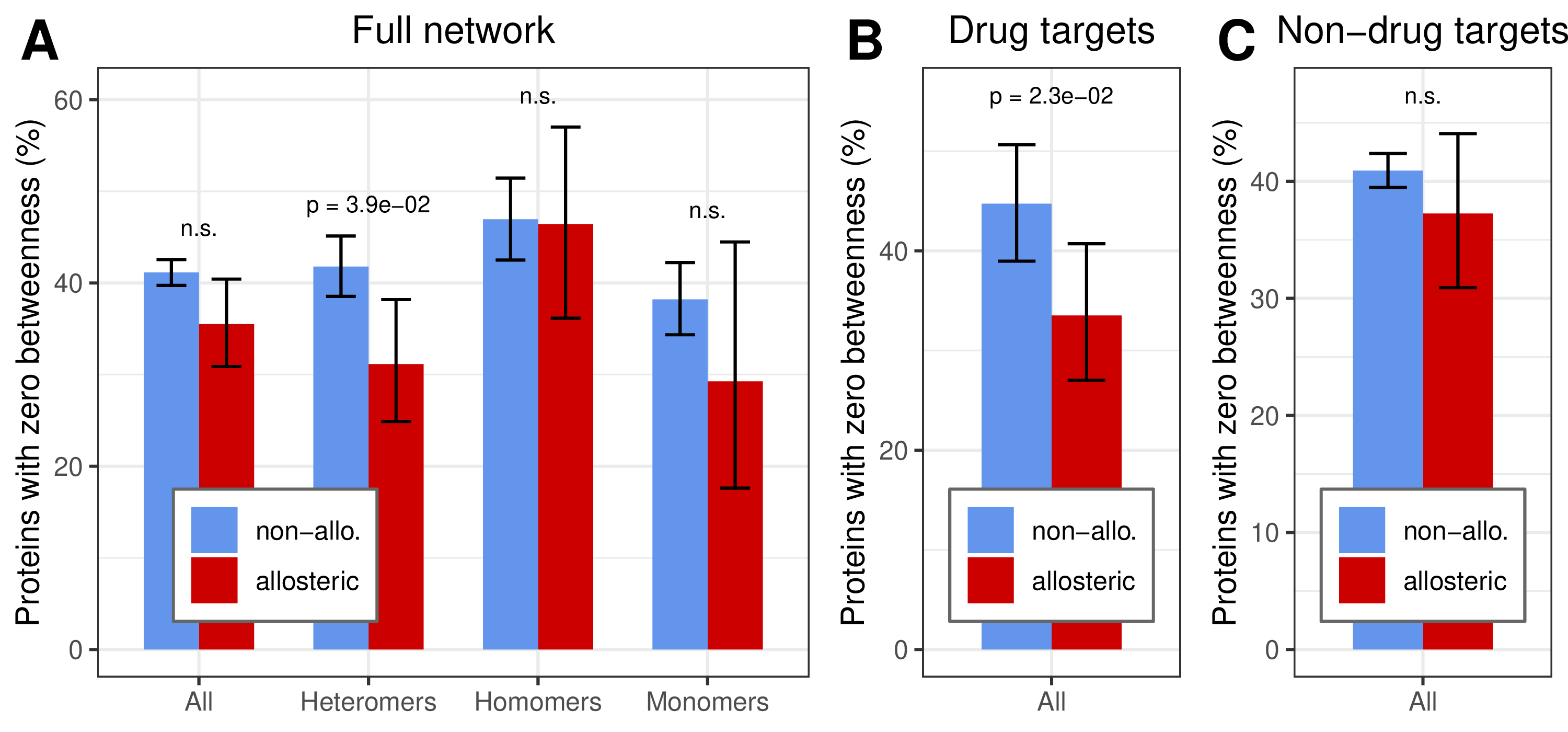

Supplement: S3 Fig — (TIF) [file pcbi.1009806.s003.tif]

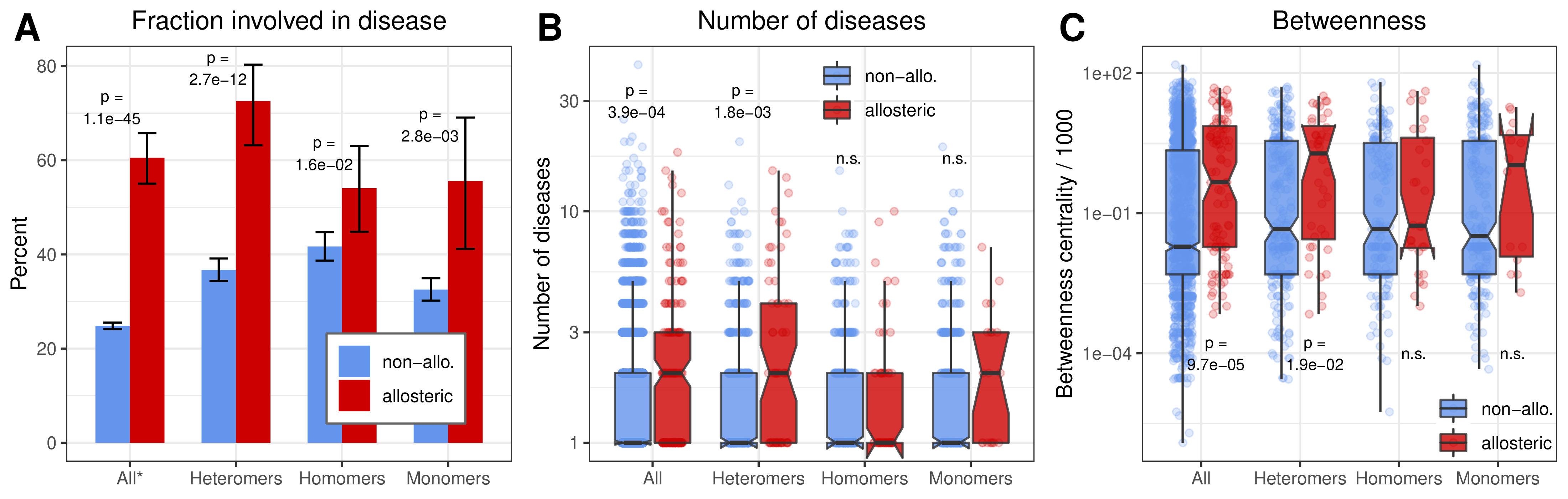

Supplement: S4 Fig — (TIF) [file pcbi.1009806.s004.tif]

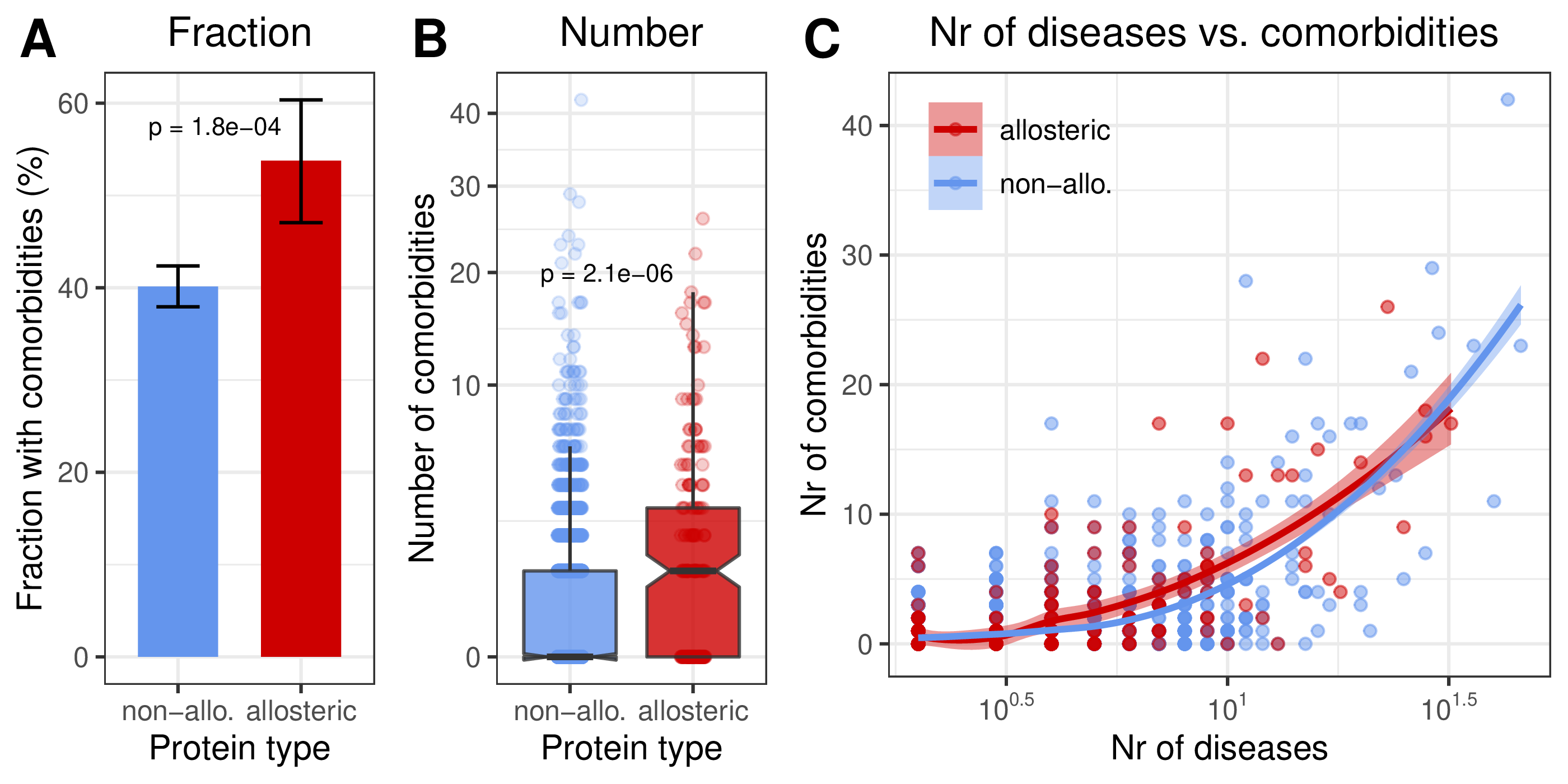

Supplement: S5 Fig — A) A significantly higher fraction of allosteric proteins is involved in known comorbidities than in non-allosteric proteins (p—tests of proportions). Only proteins associated with minimum two diseases were included. B) The number of comorbidities per protein is higher in allosteric proteins (p—Wilcoxon test). C) The relationship between the number of diseases and number of comorbidities is not qualitatively different in the two protein types, indicating that primarily the higher number diseases per protein is responsible for the higher number of comorbidities in allosteric proteins. (TIF) [file pcbi.1009806.s005.tif]

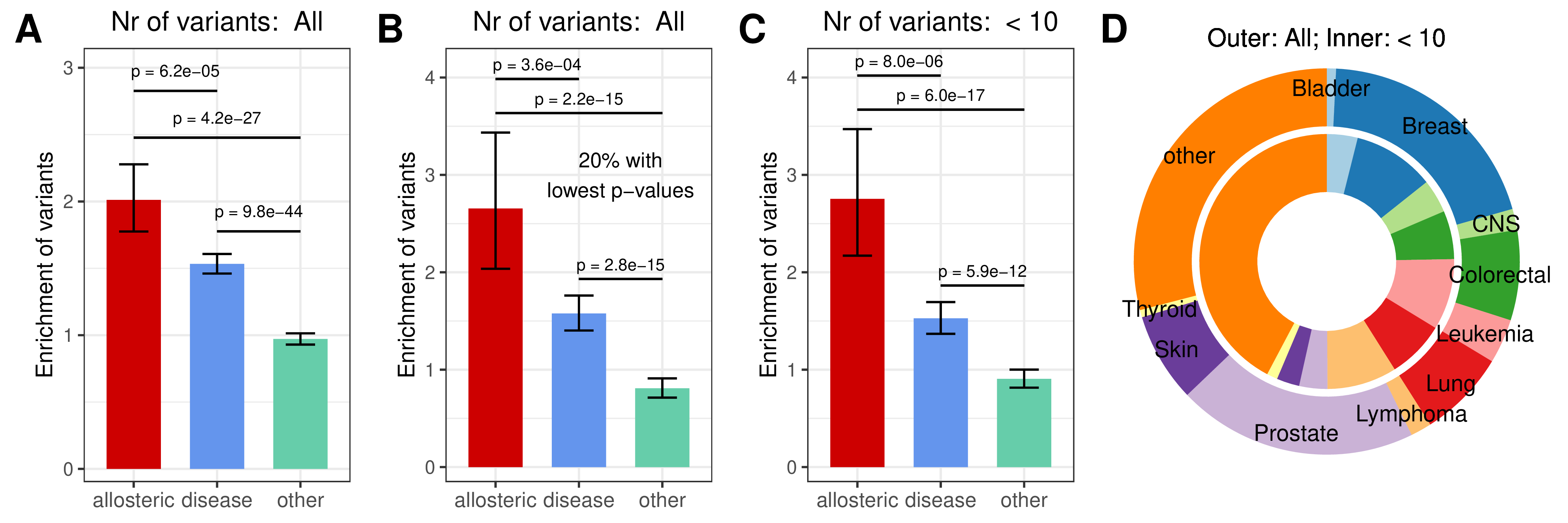

Supplement: S6 Fig — (TIF) [file pcbi.1009806.s006.tif]

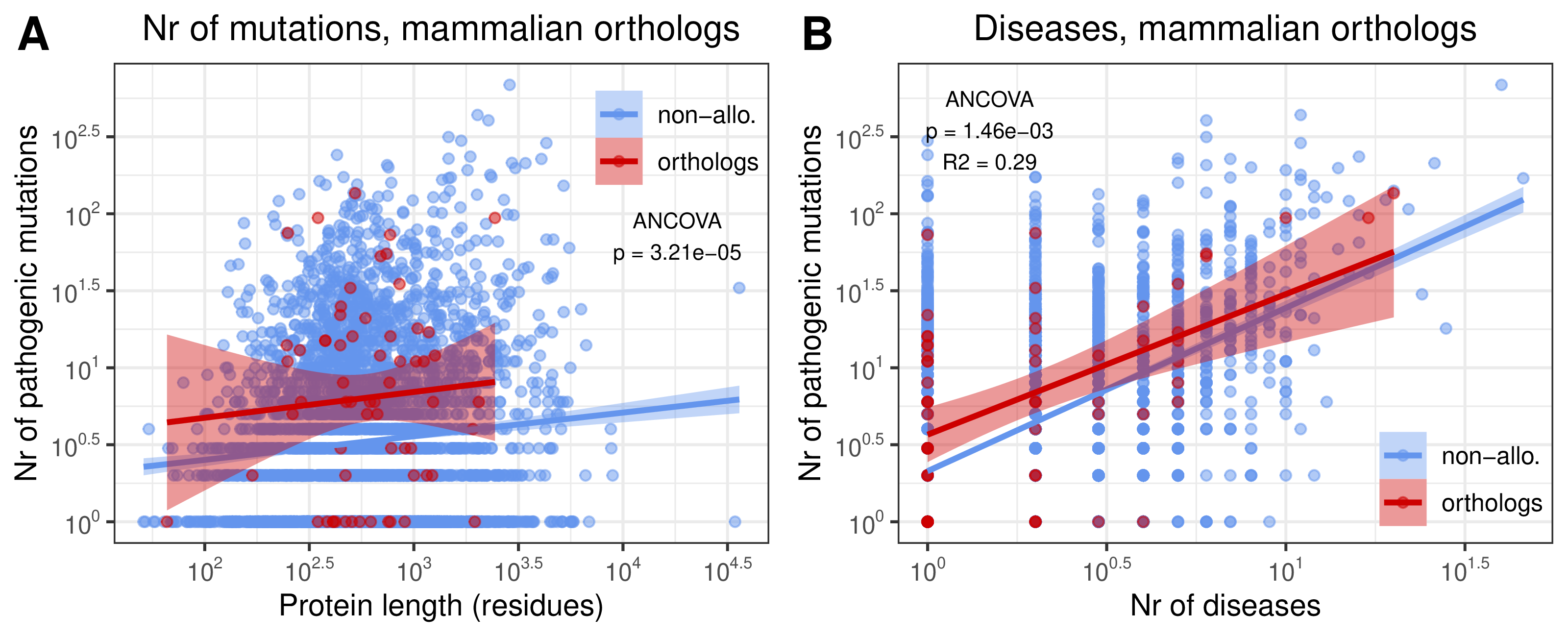

Supplement: S7 Fig — Human orthologs of mammalian allosteric proteins have similarly high number of pathogenic mutations as human allosteric proteins (A), and the relationship between the number of mutations and number of diseases is also qualitatively similar (B). (TIF) [file pcbi.1009806.s007.tif]

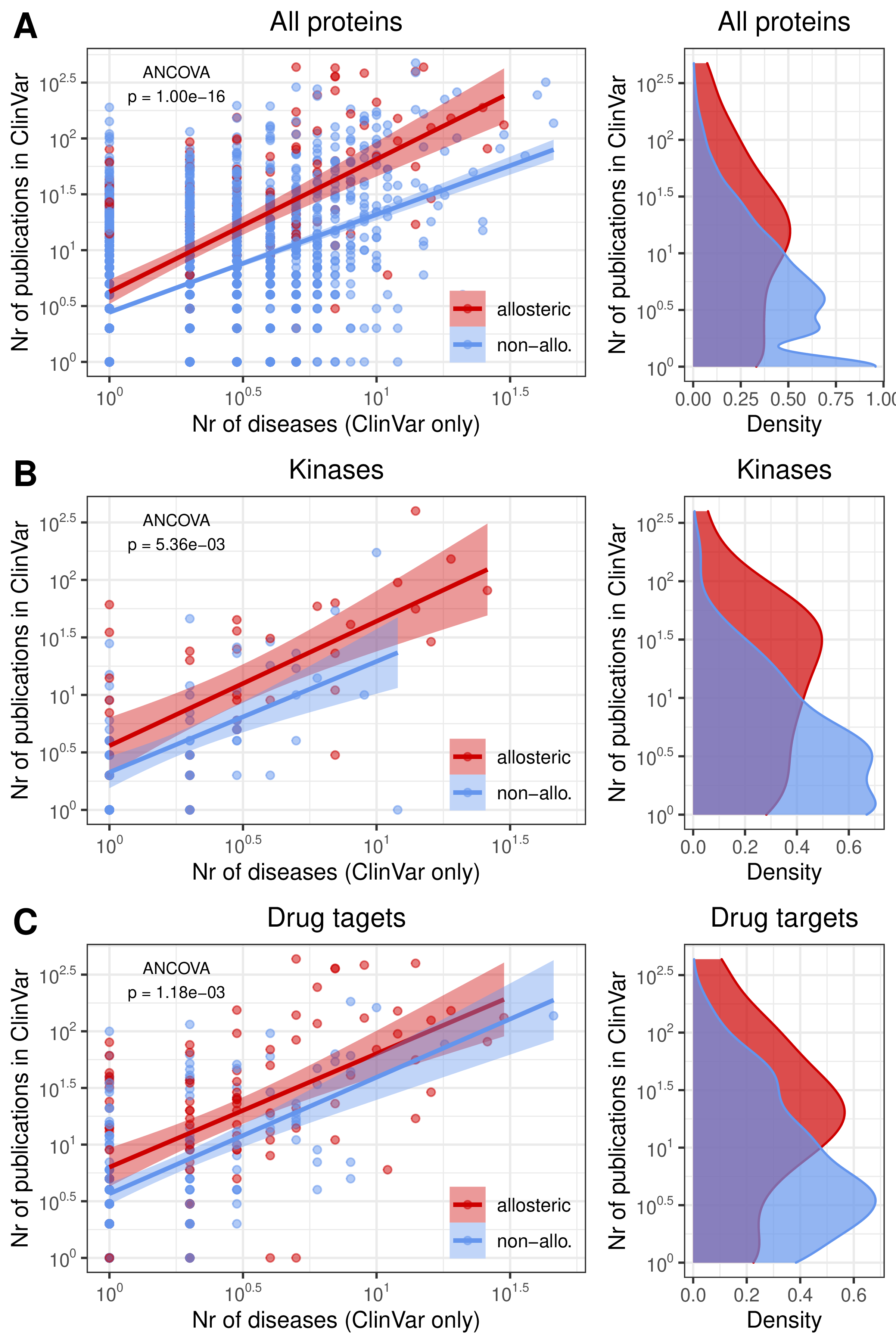

Supplement: S8 Fig — A) All proteins. B) Kinases C) Drug targets. The number of diseases was calculated using ClinVar only. The number of publications for each protein was calculated by summing all citation IDs of their “pathogenic” and “likely pathogenic” mutations, using the var_citations.txt file provided by ClinVar. (TIF) [file pcbi.1009806.s008.tif]

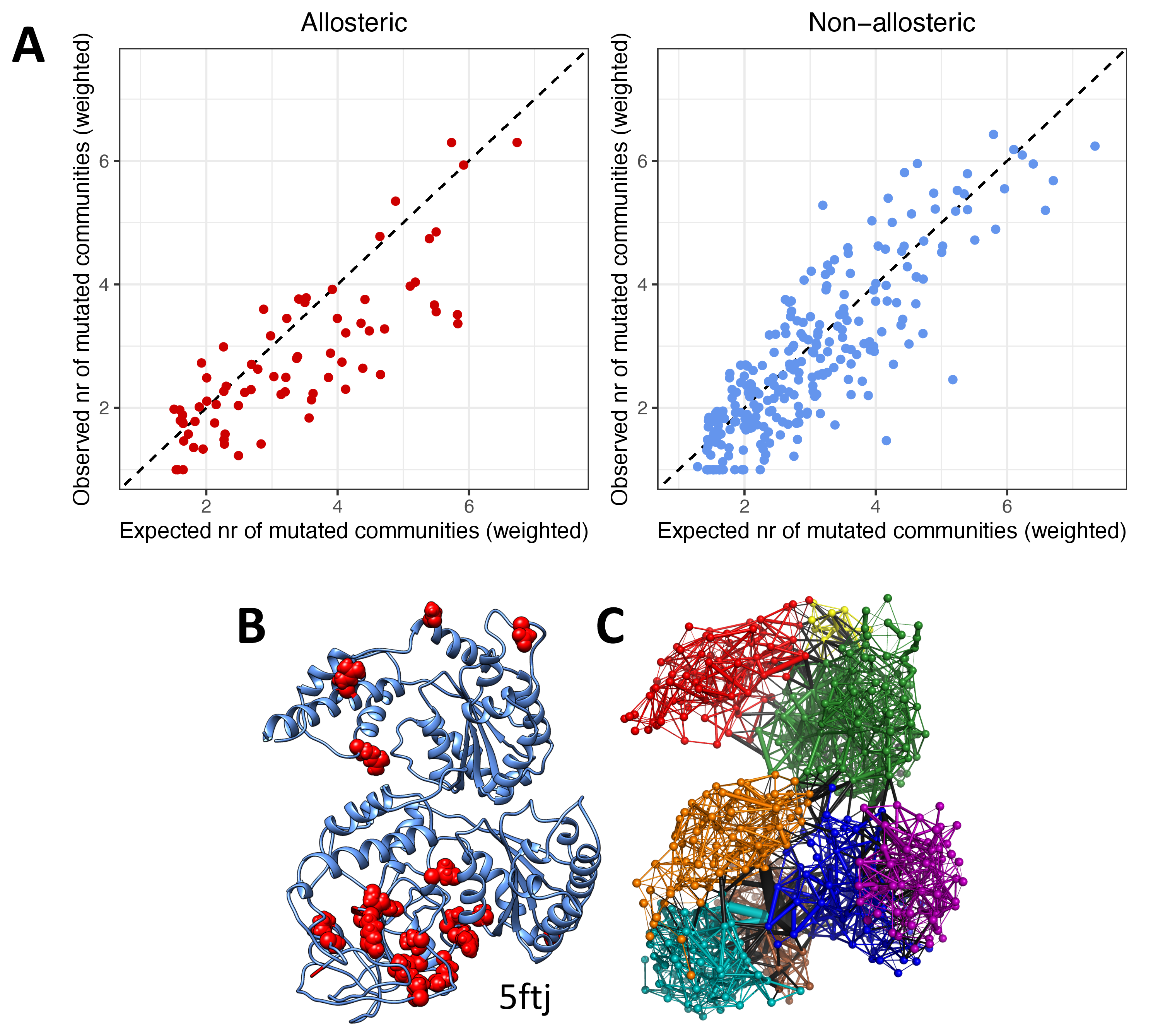

Supplement: S9 Fig — A) In allosteric proteins the difference between the expected and observed number of mutated communities is significantly higher than in non-allosteric proteins (p = 9.37e-04, ANCOVA), indicating stronger pathogenicity of mutations in allosteric proteins. B and C) Location of pathogenic mutations and communities in a monomeric unit of the Transitional Endoplasmic Reticulum ATPase (PDB ID: 5ftj). (TIF) [file pcbi.1009806.s009.tif]

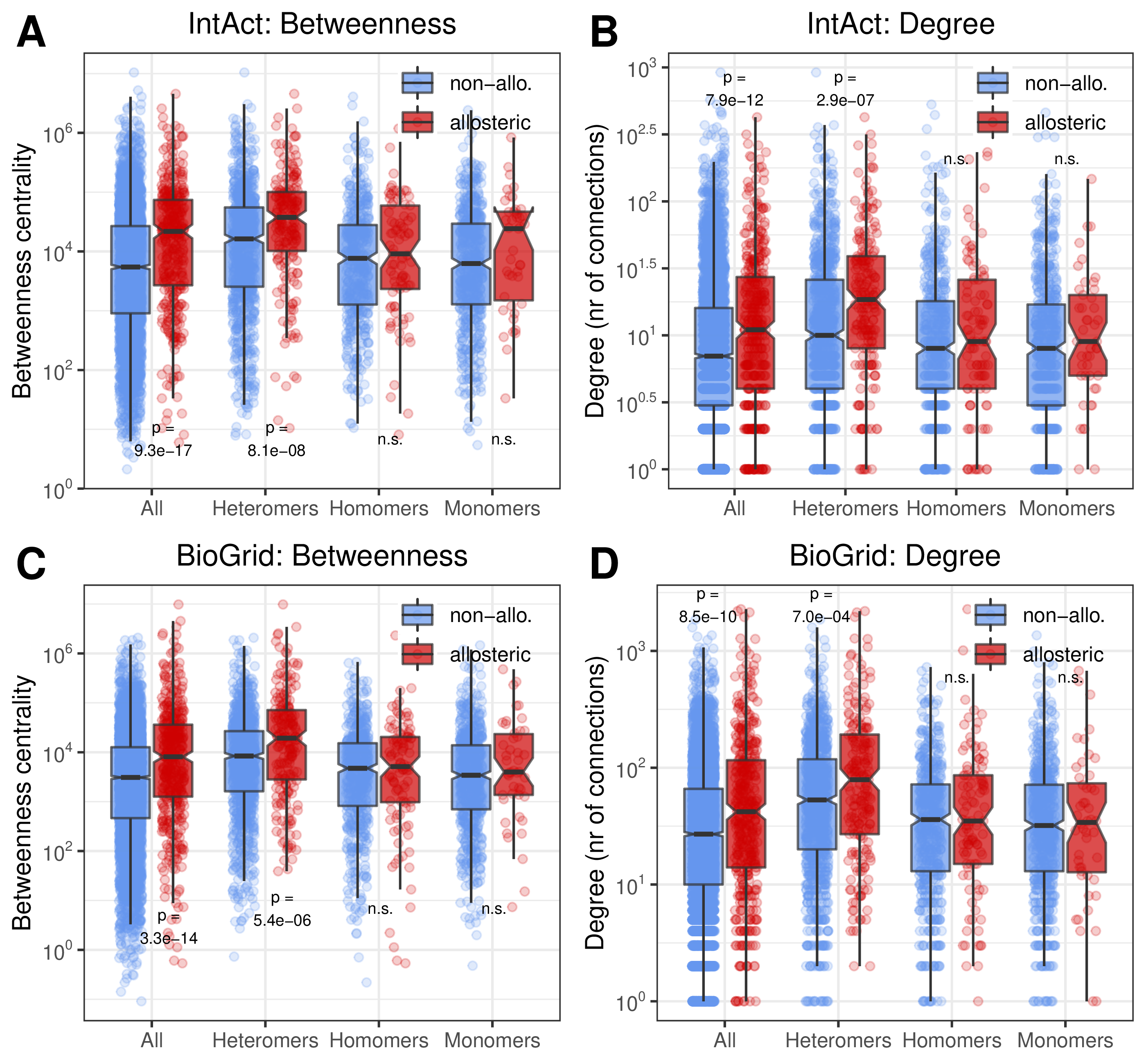

Supplement: S10 Fig — A) Betweenness centralities based on IntAct PPIs. Note that the ‘All’ category includes also the proteins where quaternary structure is not known (i.e. are not present in the PDB). B) Degree centralities (number of interactions) based on IntAct PPIs. C-D) Betweenness and degree centralities based on BioGrid PPIs. (TIF) [file pcbi.1009806.s010.tif]

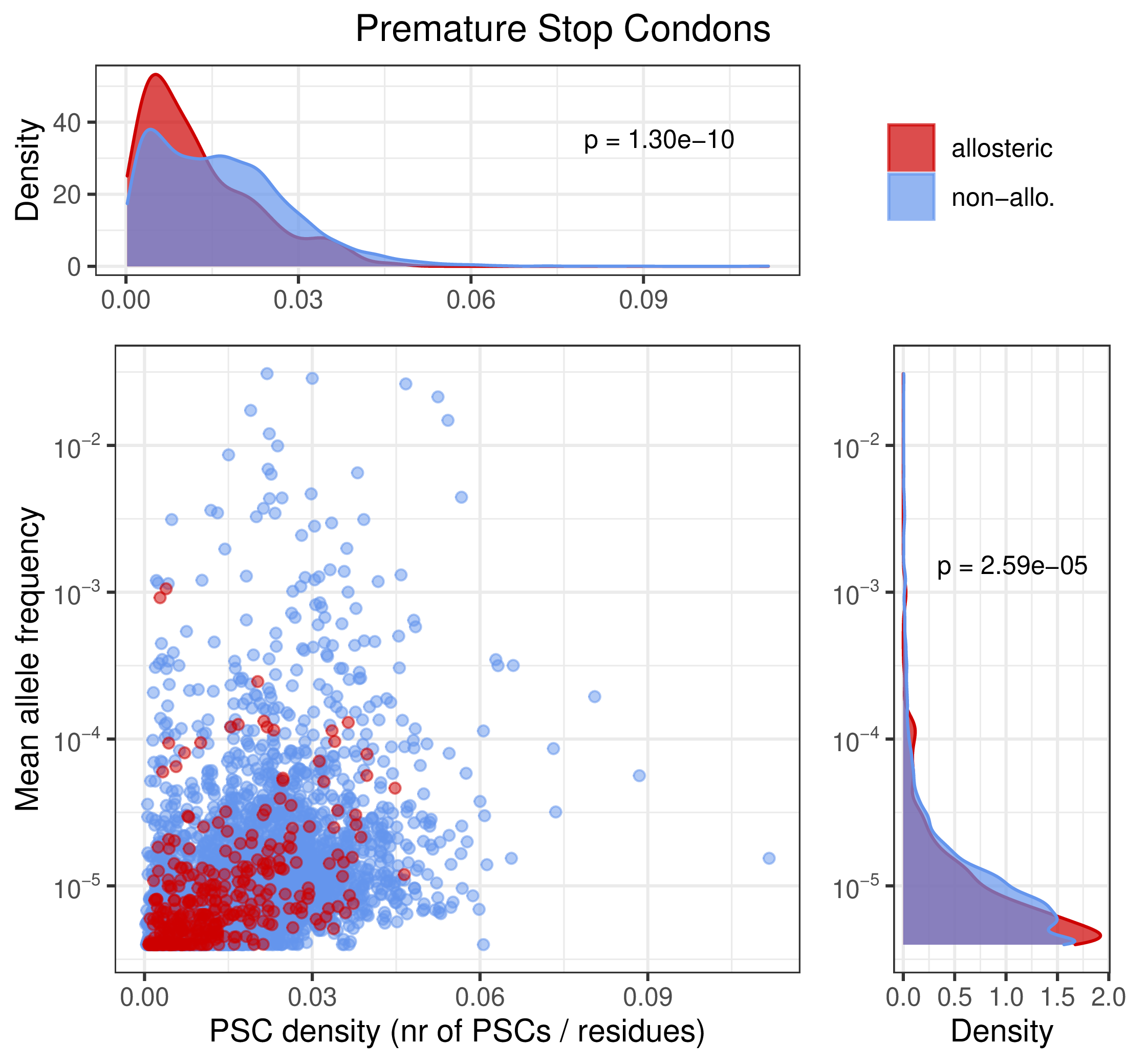

Supplement: S11 Fig — Allosteric proteins are characterized by somewhat lower PSC densities and allele frequencies than non-allosteric ones. (TIF) [file pcbi.1009806.s011.tif]
